# Identification of *Plasmodium malariae*, a Human Malaria Parasite, in Imported Chimpanzees

**DOI:** 10.1371/journal.pone.0007412

**Published:** 2009-10-12

**Authors:** Toshiyuki Hayakawa, Nobuko Arisue, Toshifumi Udono, Hirohisa Hirai, Jetsumon Sattabongkot, Tomoko Toyama, Takafumi Tsuboi, Toshihiro Horii, Kazuyuki Tanabe

**Affiliations:** 1 Laboratory of Malariology, International Research Center of Infectious Diseases, Research Institute for Microbial Diseases, Osaka University, Suita, Osaka, Japan; 2 Department of Molecular Protozoology, Research Institute for Microbial Diseases, Osaka University, Suita, Osaka, Japan; 3 The Chimpanzee Sanctuary Uto, Sanwa Kagaku Kenkyusho, CO., LTD, Uki, Kumamoto, Japan; 4 Primate Research Institute, Kyoto University, Inuyama, Aichi, Japan; 5 Department of Entomology, Armed Forces Research Institute of Medical Sciences, Bangkok, Thailand; 6 Cell-Free Science and Technology Research Center, Ehime University, Matsuyama, Ehime, Japan; INSERM U567, Institut Cochin, France

## Abstract

It is widely believed that human malaria parasites infect only man as a natural host. However, earlier morphological observations suggest that great apes are likely to be natural reservoirs as well. To identify malaria parasites in great apes, we screened 60 chimpanzees imported into Japan. Using the sequences of small subunit rRNA and the mitochondrial genome, we identified infection of *Plasmodium malariae*, a human malaria parasite, in two chimpanzees that were imported about thirty years ago. The chimpanzees have been asymptomatic to the present. In Japan, indigenous malaria disappeared more than fifty years ago; and thus, it is most likely inferred that the chimpanzees were infected in Africa, and *P. malariae* isolates were brought into Japan from Africa with their hosts, suggesting persistence of parasites at low level for thirty years. Such a long term latent infection is a unique feature of *P. malariae* infection in humans. To our knowledge, this is the first to report *P. malariae* infection in chimpanzees and a human malaria parasite from nonhuman primates imported to a nonendemic country.

## Introduction

Malaria is a major infectious disease prevalent in most tropical and subtropical areas in the world. Malaria parasites, genus *Plasmodium*, infect all classes of terrestrial vertebrates (i.e. mammals, birds, and reptiles)[1]. Of them, the four classical human malaria parasites, *Plasmodium falciparum*, *Plasmodium vivax*, *Plasmodium malariae*, and *Plasmodium ovale*, are widely believed to infect only man as a natural host. However, earlier studies described several human malaria parasite-like species from great apes: *Plasmodium reichenowi*, a *P. falciparum*-like parasite in chimpanzees, *Plasmodium schwetzi*, a *P. vivax*/*P. ovale*-like parasite in chimpanzees and gorillas, and *Plasmodium rodhaini*, a *P. malariae*-like parasite in chimpanzees [2], [3]. Determination of host specificity or host range of human malaria parasites is of great importance not only for further understanding the parasite biology but also for better malaria control. Surveys of malaria parasites in great apes are thus required. Besides, the investigation of malaria infection in great apes should be helpful for the primates' health and biodiversity conservation efforts.

The *Plasmodium* species reportedly identified as great ape malaria parasites were described in the early 20th century [3]. These previous studies were ambiguous as to whether natural infections of great apes are due to human malaria parasite-like species or to human malaria parasites. Recently, evidence of human infections of *Plasmodium knowlesi*, an Asian simian malaria parasite, is accumulating with the aid of current molecular diagnostic tools [4]–[7]. Prior studies on great ape malaria parasites may consequently be supported or disputed by new surveys using molecular diagnosis. Of the great ape parasites, *P. reichenowi* is the only species that has been confirmed to be close to but independent from *P. falciparum* at the molecular level [8]. Other malaria parasites from great apes await species identification using molecular analysis and phylogenetic relationship to human malaria parasites.

Recently, a new species, *Plasmodium gaboni*, has been identified from chimpanzees, and defined as a close relative of *P. falciparum* [9]. *P. ovale* has also been identified in chimpanzees in Africa, suggesting that *P ovale* can infect chimpanzees as a natural host [10].

Here, we report malaria parasites in two chimpanzees imported into Japan thirty years ago. The parasites isolated from these chimpanzees were identified as *P. malariae* based on two gene markers. The infections have been asymptomatic to the present, and have persisted for about thirty years. This study also indicates that human malaria parasite has been maintained in nonhuman primates in a nonendemic country, which has significance to public health issues.

## Results

Blood samples of 60 chimpanzees (*Pan troglodytes*) imported into Japan were examined. First, molecular diagnosis for the presence of malaria parasites was carried out using polymerase chain reactions (PCRs) that specifically amplify the mitochondrial genome and nuclear-encoded small subunit (SSU) rRNA gene of all known malaria parasites. PCR diagnosis yielded malaria positives in two chimpanzees, Takaboh and Oumu.

Takaboh is a male chimpanzee (*Pan troglodytes verus*), assumed to have been born in 1978. He was imported into Japan from the Republic of Sierra Leone in April 1980. His blood samples were collected during routine health examinations in January 2003 and in September 2008. PCR diagnosis was positive on both occasions. Oumu is a female chimpanzee (*Pan troglodytes verus*), assumed to have been born in 1976. She was imported into Japan from Africa in March 1977. There is no record of her country of origin. It is likely that Oumu came from West Africa because she belongs to *P. t. verus*, a subspecies living in the western part of Africa. Her blood was obtained during routine health examination in June 2003. Importantly, Takaboh and Oumu have shown no symptoms of malaria to the present while in Japan.

**Figure 1:**
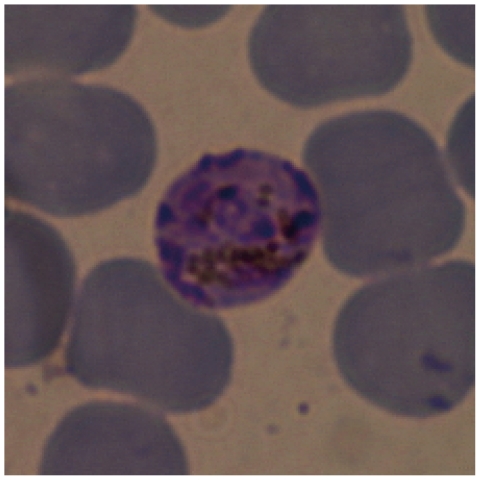
Giemsa-stained thin blood smear showing an immature schizont.

Microscopic observations of Giemsa-stained thin blood smears were done for specimens available for Takaboh and Oumu. Blood smears of Takaboh were prepared in August 2000, January and June 2003, and September 2008, and those of Oumu were made in October 1986, October 1996, and June 2003. We were able to detect, at one instance, one erythrocyte infected with malaria parasite in a Takaboh specimen obtained in September 2008 (Figure 1), but detection was unsuccessful from the other samples. The parasite shows the morphology of an immature schizont having irregular masses of chromatin, resembling *P. malariae*. The typical band-form of *P. malariae* could not be seen (Figure 1).

**Figure 2:**
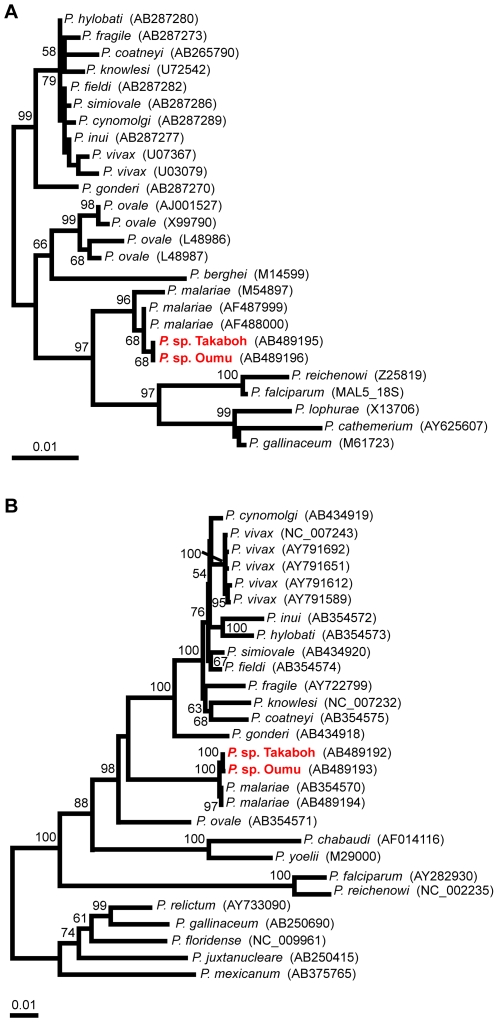
Phylogenetic trees of SSU rRNA gene (A) and mitochondrial genome (B). The numbers on the phylogenetic tree represent bootstrap values based on 1000 replications. GenBank accession numbers are in brackets.

To identify the malaria parasite species from Takaboh and Oumu, the complete nucleotide sequence of the mitochondrial genome and the near-complete SSU rRNA gene sequence were obtained from these chimpanzee parasites. We also obtained an additional sequence of the mitochondrial genome from human *P. malariae* (Thailand isolate) because only one sequence (Uganda I isolate; GenBank accession number AB354570) was available. The sequences obtained were used for the construction of phylogenetic trees together with those from diverse malaria parasite species including primate, rodent, bird, and reptile parasites (Figure 2). In the phylogenetic tree of the SSU rRNA gene, both parasites of Takaboh and Oumu are clearly located within the cluster of human *P. malariae* isolates. This indicates that the two chimpanzee parasites belong to *P. malariae* (Figure 2A). Likewise, the phylogenetic tree of the mitochondrial genome represents clustering of the chimpanzee parasites with human *P. malariae* isolates. A closer look shows that the parasites of Takaboh and Oumu make a small sub-cluster and seem to be separated from the human *P. malariae* isolates (Figure 2B). This separation, however, does not indicate that the chimpanzee parasite is an independent species different from *P. malariae*. The genetic difference of the mitochondrial genome between the chimpanzee parasites and human *P. malariae* isolates should be regarded as an intraspecific variation (polymorphism): that is, the p-distances (the proportion of nucleotide sites at which two sequences being compared are different) between the parasites of Takaboh and Oumu and the human *P. malariae* isolates are at most 0.0023, smaller than the p-distance between the most distantly related isolates of *P. vivax* (at least 0.0026 is the observable polymorphism range in *P. vivax* populations; the calculation was based on the sequences in ref. 14; see also Figure 2B). We, therefore, conclude that the parasites from Takaboh and Oumu are isolates of *P. malariae*.

## Discussion

The present finding of *P. malariae* isolates in two chimpanzees reinforces that *P. malariae* is able to infect chimpanzees as a natural host. Takaboh and Oumu were imported into Japan about thirty years ago at one or two years of age. In the mainland of Japan, there has been no indigenous malaria for more than 50 years [11]. These facts imply that the two chimpanzees were infected with *P. malariae* in Africa before their importation, and that *P. malariae* infection has persisted for about 30 years in the two chimpanzees. In addition, both Takaboh and Oumu have been asymptomatic to the present, and parasite densities were extremely low in their bloods. These circumstances are consistent with a long term latent infection, a unique feature of human *P. malariae* infection [12], [13]. *P. malariae* therefore appears to have a similar course of infection in chimpanzees as in humans. A much larger survey in wild chimpanzees in Africa would present more details about *P. malariae* infection in chimpanzees.

It is known that, in humans, *P. malariae* infrequently recrudesces after tens of years of dormancy [13]. Takaboh and Oumu are currently under careful observation for such latency. One may argue that malaria parasites have been transmitted between chimpanzee hosts. But despite that Takaboh and Oumu currently live in the same facility, their parasites have distinct sequences of SSU rRNA and mitochondrial genome. Furthermore, since our PCR method can detect malaria parasites at very low parasite density (as few as 1 parasite/sample using PgeneralF3s and PgeneralR1s primers; see Materials and Methods; data not shown), more infection of other chimpanzees should have been detected. Thus, it is unlikely that the parasites have been transmitted among them. However, given the public health concern/issues, malaria monitoring may be proposed as a part of routine health examination for all captive chimpanzees.

*P. rodhaini* was previously described as a *P. malariae*-like quartan malaria parasite in chimpanzees [3], [13]. Experimental transfer of *P. rodhaini* from chimpanzees to humans was successful by the inoculations of parasitized blood [3]. In addition, splenectomized chimpanzees are susceptible to *P. malariae* infection [3], [13]. These experiments lead to note that *P. rodhaini* is synonymous with *P. malariae*. Our finding is consistent with this note. However, further findings from malaria survey in great apes are definitely needed to conclude that *P. rodhaini* is a variant of *P. malariae*.

Human malaria parasites have been widely supposed to be found only in humans; hence nonhuman primates are not included in the target for malaria control. The present finding necessitates a survey of human malaria parasites in nonhuman primates. In this context, it should be remembered that two New World monkey parasites, *Plasmodium brasilianum* and *Plasmodium simium*, are known as *P. malariae*-like and *P-vivax*-like parasites, respectively [3], [13]. Evolutionary analysis represents that *P. brasilianum* and *P. simium* are very closely related to *P. malariae* and *P. vivax*, respectively [14], [15]. Thus, both New World monkey parasites might be actually human parasites infected New World monkeys. In addition to chimpanzees, the New World monkey may therefore be a subject of malaria screening for public health.

Considering that great apes are endangered animals today [16], this study also has significance in their conservation biology. Further surveys may reveal the current situation of malaria infection in great apes, and help to assess risk of malaria in their life.

## Materials and Methods

### Chimpanzee samples

The chimpanzees are being kept in the Chimpanzee Sanctuary Uto, Kumamoto (n = 55) and Primate Research Institute, Kyoto University, Aichi (n = 5). During routine health examinations, chimpanzees were sedated with oral midazolam (1 mg/kg) or droperidol (0.2 mg/kg), and their bloods were collected under anesthesia by ketamine hydrochloride (7 mg/kg) or a combination of ketamine hydrochloride (3.5 mg/kg) and medetomidine hydrochloride (0.035 mg/kg). This study was approved by Research Institute for Microbial Diseases, Osaka University; Primate Research Institute, Kyoto University; and the Chimpanzee Sanctuary Uto. All animal work has been conducted according to the following guidelines: Guide for the Care and Use of Laboratory Primates, 2nd edn. (Primate Research Institute, Kyoto University), and Guideline for Care of Chimpanzees (the Chimpanzee Sanctuary Uto). Genomic DNA of blood samples was extracted using QIAamp DNA mini kit (Qiagen, Hilden, Germany).

### SSU rRNA sequences

Genomic and nested PCRs using primers (PlaSSU5, PlaSSU3r, SSUF1, and SSUR1) were performed following methods reported in ref. 17. Briefly, genomic PCR conditions were as follows: denaturation at 95°C for 2 min followed by 30 cycles of 95°C for 15 s, 60°C for 30 s, 68°C for 5 min, and extension at 68°C for 10 min, and the nested PCR conditions were as follows: denaturation at 95°C for 2 min followed by 30 cycles of 95°C for 15 s, 59.3°C for 30 s, 68°C for 5 min, and extension at 68°C for 10 min [17]. In addition, new primers [PmSSUF2 (5′-TCTCAAAGATTAAGCCATGCAAGTG-3′), PmSSUR7 (5′-TTCACCGACGGAAACCTTGTTAC-3′), PmSSUF3 (5′-TTAAGCCATGCAAGTGAAAGTATATG-3′), and PmSSUR2 (5′-TTAAAAGATAGGATTTACGATTTTTC-3′)] were designed. PCR reactions using new primers (PmSSUF2 and PmSSUR7) were performed with 5 pmol of each primer and 1 µl of extracted genomic DNA solution in a total volume of 20 µl containing PrimeSTAR Max DNA polymerase (Takara, Otsu, Shiga, Japan). Gene Amp PCR system 9700 (Applied Biosystems, Foster City, CA) was used to generate the following conditions: 35 cycles of 98°C for 10 s, 55°C for 5 s, and extension at 72°C for 3 min. The nested PCR were performed with 10 pmol of each new primer (PmSSUF3 and PmSSUR2) and 2 µl of PCR product in a total volume of 50 µl. The nested PCR conditions were as follows: 30 cycles of 98°C for 10 s, 55°C for 5 s, and extension at 72°C for 3 min. The PCR products obtained were cloned into pCR-Blunt II-TOPO vector according to the manufacture's instructions (Invitrogen, Carlsbad, CA, USA). The plasmids containing the SSU rRNA genes were prepared using QIAGEN Plasmid Mini kit (Qiagen), and then subjected to sequencing using an ABI 3130 genetic analyzer (Applied Biosystems).

### Complete mitochondrial genome sequences

To amplify the mitochondrial genome sequences of chimpanzee parasites, the genomic PCRs using primers (PgeneralF2s, PgeneralR2, PgeneralF3s, and PgeneralR1s) were performed following methods reported in ref. 18. PCR conditions were as follows: denaturation at 93°C for 1 min followed by 40 cycles of 93°C for 20 s, 60°C for 1 min, 72°C for 3 or 5 min, and extension at 72°C for 10 min [18]. The nested PCRs were carried out using the nested primers [CP69F1 (5′-ATTTAGCGTGTATTGTTGCCTTGTAC-3′), PgeneralR2 (ref. 18), PvmtF1001 (5′-CATGCAGGACGGAGATTACCCGA-3′), and PgeneralR1s (ref. 18)] to obtain sufficient amount of PCR products for sequencing. The nested PCR reactions were performed with 10 pmol of each primer and 2 µl of PCR product in a total volume of 50 µl containing 400 µM dNTPs and 1 unit of LA-Taq DNA polymerase (Takara) in PCR buffer containing 2.5 mM MgCl_2_. The nested PCR conditions were as follows: denaturation at 93°C for 1 min followed by 20 cycles of 93°C for 20 s, 60°C for 1 min, 72°C for 3 or 5 min, and extension at 72°C for 10 min.

In addition, we amplified mitochondrial genome sequences from one human *P. malariae* isolate (PVMS1229, Thailand). The genomic PCRs were performed with the primers [PgeneralF3s (ref. 18), PgeneralR1s (ref. 18), PmF1 (5′-CTAGCATGGGACTAAAAAATGTTATG-3′), PmR3 (5′-CTGTATCGTACCCTAAAGGATTAG-3′), PmF3 (5′-AATTATGGAGTGGATGGTGTTTTAG-3′), and PmR1 (5′-AGAAGTTAATATCTGGAAGCGTCTG-3′)] and 2 µl of extracted genomic DNA solution under the following conditions: denaturation at 93°C for 1 min followed by 40 cycles of 93°C for 20 s, 59 or 60°C for 1 min, 72°C for 3 min, and extension at 72°C for 10 min. The nested PCRs were carried out using the nested primers [PvmtF1001 (5′-CATGCAGGACGGAGATTACCCGA-3′), PgeneralR1s (ref. 18), PmF2 (5′-TTAAGCCCTTTTTACCATACAAGAG-3′), PmR4 (5′-ATCTTTTTATAGTTGGATCACTTACAG-3′), PmF4 (5′-TTACAGCTTTTATAGGTTATGTTTTAC-3′), and PmR2 (5′-GTATCGTAAACGGTCCTAAGGTAG-3′)] to obtain sufficient amount of PCR products for sequencing. The nested PCR conditions were as follows: denaturation at 93°C for 1 min followed by 20 cycles of 93°C for 20 s, 57 or 60°C for 1 min, 72°C for 3 min, and extension at 72°C for 10 min.

PCR products were purified using the QIA quick PCR purification Kit (Qiagen) and directly sequenced on an ABI 3130 genetic analyzer (Applied Biosystems).

### Phylogenetic analysis

Maximum Likelihood trees of SSU rRNA gene and mitochondrial genome were inferred using PAUP 4.0 b [19] based on the GTR+Γ+I model from the selected 1419 sites and the GTR+Γ+I model from the selected 5837 sites, respectively. To construct trees, primate parasites (*Plasmodium falciparum*, *Plasmodium vivax*, *Plasmodium malariae*, *Plasmodium ovale*, *Plasmodium reichenowi*, *Plasmodium hylobati*, *Plasmodium knowlesi*, *Plasmodium cynomolgi*, *Plasmodium coatneyi*, *Plasmodium fieldi*, *Plasmodium fragile*, *Plasmodium gonderi*, *Plasmodium inui*, and *Plasmodium simiovale*), rodent parasites (*Plasmodium berghei*, *Plasmodium chabaudi*, and *Plasmodium yoelii*), bird parasites (*Plasmodium cathemerium*, *Plasmodium gallinaceum*, *Plasmodium juxtanucleare*, *Plasmodium lophurae*, and *Plasmodium relictum*), and reptile parasites (*Plasmodium floridense* and *Plasmodium mexicanum*) were used. The complete or nearly complete sequences of mitochondrial genome and SSU rRNA gene were obtained from the NCBI Web site (http://www.ncbi.nlm.nih.gov/; see also refs. 17 and 18).
